# Associação entre o Índice de Aterosclerose Inflamatória e a Doença Hepática Gordurosa Não Alcoólica: NHANES 2017–2020

**DOI:** 10.36660/abc.20260068

**Published:** 2026-10-21

**Authors:** Yun Jiang, Jingying Xie, Ying Dong

**Affiliations:** 1 Department of Hepatobiliary and Pancreatic Surgery II Ningbo Medical Centre Lihuili Hospital Zhejiang Province Ningbo China Department of Hepatobiliary and Pancreatic Surgery II, Ningbo Medical Centre Lihuili Hospital, Zhejiang Province, Ningbo – China

**Keywords:** Hepatopatia Gordurosa não Alcoólica, Inquéritos Nutricionais, Estudos Transversais

## Abstract

**Fundamento:**

A prevalência da doença hepática gordurosa não alcoólica (DHGNA) está aumentando globalmente e está intimamente associada ao metabolismo lipídico desregulado e à inflamação. No entanto, a relação entre a DHGNA e o índice de aterosclerose inflamatória (IAI), que reflete esses processos, ainda não está clara.

**Objetivos:**

Investigar a associação entre IAI e DHGNA em uma amostra representativa de adultos dos EUA.

**Métodos:**

Este estudo transversal incluiu 5.309 adultos do banco de dados NHANES dos EUA. A regressão logística ponderada multivariada foi usada para avaliar a associação entre IAI e DHGNA. Splines cúbicos restritos exploraram relações não lineares e a análise ROC avaliou o desempenho diagnóstico.

**Resultados:**

Um total de 5.309 participantes foram incluídos, dos quais 37,6% apresentavam DHGNA. A obesidade e as anormalidades metabólicas foram mais pronunciadas no grupo com DHGNA, e os níveis de IAI foram significativamente maiores (p < 0,001). A análise multivariada demonstrou que o IAI estava associado de forma independente e positiva à DHGNA (OR = 1,538, IC 95%: 1,421–1,665). A análise RCS indicou uma relação não linear (P para não linearidade = 0,0473), com um ponto de inflexão em IAI = 0,145. Quando IAI < 0,145, o risco aumentou acentuadamente (OR = 2,341, IC 95%: 2,037–2,690), enquanto quando IAI ≥ 0,145, o aumento foi atenuado (OR = 1,399, IC 95%: 1,263–1,548). As análises de subgrupos mostraram resultados consistentes, sem interações significativas entre os subgrupos. A análise ROC demonstrou que o IAI apresentou o melhor desempenho diagnóstico (AUC = 0,722), superando significativamente os demais indicadores (todos com p < 0,001).

**Conclusões:**

Entre adultos nos EUA, o IAI demonstra uma associação forte, positiva e não linear com a DHGNA. Esses achados sugerem que o IAI é um marcador populacional promissor para DHGNA, embora estudos prospectivos sejam necessários para validação.

## Graphical abstract

**Figura Central:**
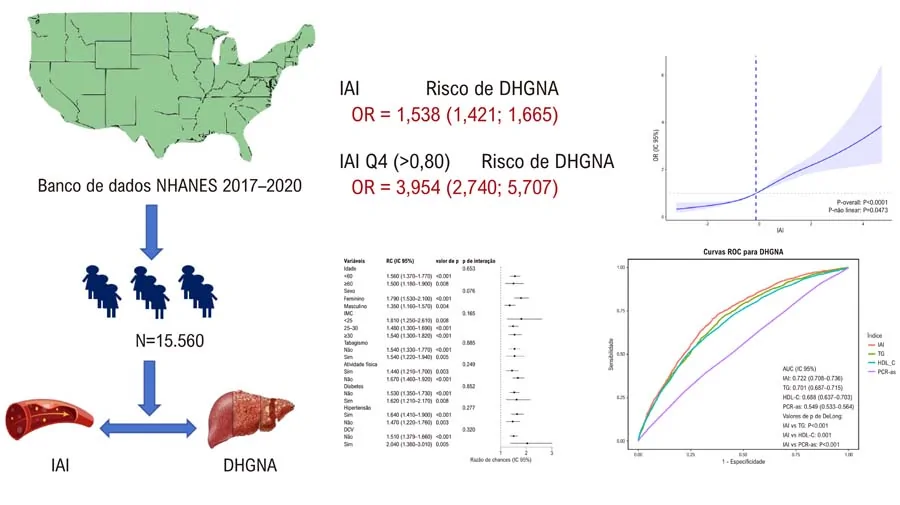
Associação entre o Índice de Aterosclerose Inflamatória e a Doença Hepática Gordurosa Não Alcoólica: NHANES 2017–2020

## Introdução

A doença hepática gordurosa não alcoólica (DHGNA) é uma das doenças hepáticas crônicas mais comuns em todo o mundo. A prevalência da DHGNA aumentou em paralelo com o aumento das taxas de obesidade, diabetes tipo 2 e síndrome metabólica, representando um grande desafio global para a saúde pública.^1-3^ A fisiopatologia da DHGNA é complexa e envolve múltiplos fatores, incluindo metabolismo lipídico desregulado, resistência à insulina, inflamação crônica de baixo grau e estresse oxidativo.^4,5^ À medida que a DHGNA progride, uma parcela dos pacientes desenvolve esteato-hepatite não alcoólica, fibrose hepática, cirrose ou carcinoma hepatocelular. A identificação precoce de indivíduos de alto risco e dos fatores de risco associados é, portanto, crucial para o manejo da doença, a estratificação de risco e o planejamento de estratégias de intervenção.

A inflamação e o metabolismo lipídico desregulado são considerados mecanismos centrais que impulsionam o início e a progressão da DHGNA.^6,7^ A inflamação crônica de baixo grau promove o acúmulo de lipídios no fígado e lesão hepatocelular por meio de citocinas pró-inflamatórias e vias mediadas pelo estresse oxidativo. Enquanto isso, a dislipidemia pode exacerbar o acúmulo de gordura hepática e o estresse metabólico. Essa interação entre inflamação e metabolismo lipídico não apenas influencia a patologia hepática, mas também pode aumentar o risco cardiovascular e metabólico sistêmico.^8,9^ Portanto, indicadores que integram informações inflamatórias e lipídicas são valiosos para estudos epidemiológicos da DHGNA.

O índice de aterosclerose inflamatória (IAI) é uma medida composta que integra parâmetros lipídicos e a carga inflamatória sistêmica.^10^ O IAI combina medições de lipídios com proteína C-reativa de alta sensibilidade (PCR-as) para quantificar a dislipidemia e a carga inflamatória sistêmica, refletindo assim o estresse metabólico geral. Valores mais altos de IAI indicam maiores anormalidades lipídicas e carga inflamatória, refletindo um aumento do estresse metabólico relacionado à inflamação e servindo como um potencial biomarcador para doenças relacionadas ao metabolismo.

Embora estudos anteriores tenham relacionado dislipidemia e estado inflamatório à DHGNA^11,12^ Poucas análises sistemáticas avaliaram índices compostos como o IAI. Portanto, avaliamos sistematicamente a associação entre o IAI e a DHGNA utilizando a amostra nacionalmente representativa do NHANES 2017–2020. Nosso objetivo foi fornecer evidências empíricas para a identificação precoce do risco e a caracterização das características metabólicas da DHGNA, além de orientar o uso potencial de marcadores relacionados à função hepática e à inflamação para a predição da doença em populações sem tumores.

## Materiais e métodos

### Coleta de dados

Este estudo realizou uma análise transversal utilizando dados dos ciclos NHANES de 2017 a 2020. O NHANES é um levantamento nacional representativo sobre saúde e nutrição nos Estados Unidos, realizado a cada dois anos. Ele coleta informações sobre o estado de saúde e nutricional dos participantes por meio de questionários, exames físicos e exames laboratoriais. A amostra inicial incluiu 15.560 participantes. Para garantir uma população de estudo adequada e dados completos, excluímos participantes com menos de 20 anos, aqueles com teste positivo para antígeno de superfície da hepatite B ou anticorpo/RNA da hepatite C, indivíduos que relataram consumo excessivo de álcool (≥ 4 a 5 doses por dia) e aqueles com dados faltantes nas covariáveis. Um total de 5.309 adultos elegíveis foram incluídos na amostra analítica final. O processo de limpeza e triagem dos dados é apresentado na Figura 1 e na Figura central.

**Figura 1:**
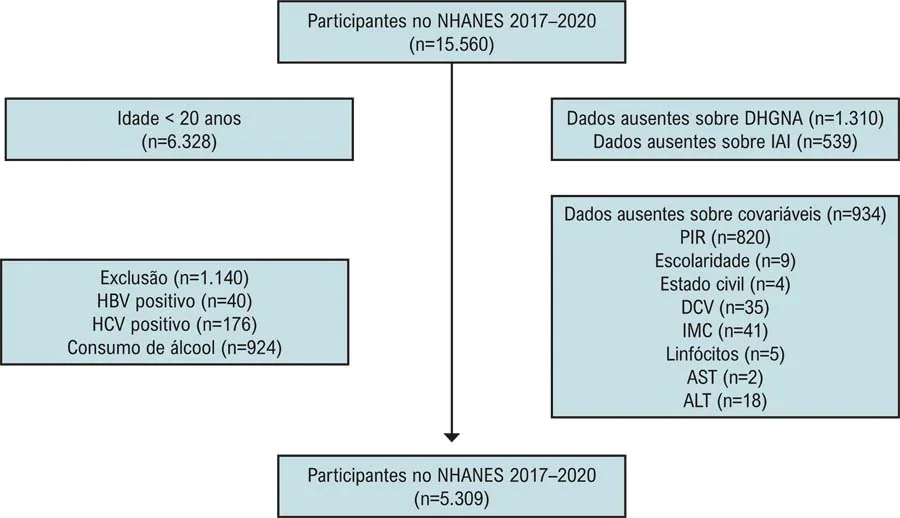
– Fluxograma da seleção de participantes do NHANES 2017–2020. IAI: índice de aterosclerose inflamatória; NHANES: Pesquisa Nacional de Saúde e Nutrição; PIR: razão de renda e pobreza; HBV: vírus da hepatite B; HCV: vírus da hepatite C; DCV: doenças cardiovasculares; ALT: alanina aminotransferase; AST: aspartato aminotransferase; IMC: índice de massa corporal.

### Definição de doença hepática gordurosa não alcoólica

A determinação da DHGNA se baseia no parâmetro de atenuação controlada (PAC) obtido por elastografia hepática transitória. De acordo com a literatura anterior,^13^ inflamatório de aterosclerose em indivíduos com valor de CAP superior a 285 dB/m foram classificados como portadores de DHGNA, enquanto todos os demais foram alocados ao grupo não-DHGNA. Esse critério fornece um método confiável e não invasivo para avaliar o acúmulo de gordura no fígado.

### Índice de aterosclerose inflamatória

O IAI é um indicador composto que reflete de forma abrangente as anormalidades do metabolismo lipídico e a carga inflamatória sistêmica, sendo utilizado para capturar a interação entre dislipidemia e inflamação sistêmica.^14^ O IAI é calculado como: IAI = log10 (triglicerídeos / colesterol HDL) × hs-CRP / 10. Valores de IAI mais altos indicam maiores anormalidades no metabolismo lipídico e uma maior carga inflamatória sistêmica.

### Covariável

Este estudo incluiu como covariáveis sexo, idade, raça/etnia, nível de escolaridade, estado civil, índice de pobreza/renda familiar (PIR), índice de massa corporal (IMC), tabagismo, hipertensão, diabetes, doença cardiovascular (DCV), nível de atividade física, alanina aminotransferase (ALT), aspartato aminotransferase (AST) e o índice de resposta inflamatória sistêmica (SIRI). O diagnóstico de diabetes baseou-se em autodeclaração, uso de insulina ou hipoglicemiantes orais e indicadores laboratoriais (glicemia de jejum ≥ 7,0 mmol/L ou HbA1c ≥ 6,5%).^15^As DCVs incluem doença arterial coronariana, angina pectoris, infarto do miocárdio, insuficiência cardíaca e acidente vascular cerebral. As informações foram obtidas do Questionário de Histórico Médico do NHANES.^16^ A hipertensão foi definida como uma pressão arterial sistólica média ≥ 140 mmHg, uma pressão arterial diastólica média ≥ 90 mmHg, ou autodeclaração de diagnóstico ou uso de medicamentos anti-hipertensivos.^17^ Os valores de ALT, AST e SIRI foram obtidos a partir das medições laboratoriais do NHANES. O SIRI foi calculado como monócitos × neutrófilos ÷ linfócitos.^18^

### Análise estatística

Este estudo levou em consideração o complexo desenho amostral do NHANES, incluindo estratificação, agrupamento e amostragem em múltiplos estágios. A ponderação da pesquisa foi aplicada para garantir a representatividade nacional e a generalização dos resultados. As características basais dos participantes foram descritas de acordo com o status de DHGNA. As variáveis contínuas foram apresentadas como média ± desvio padrão (DP) e as variáveis categóricas como frequência e porcentagem. As diferenças entre os grupos foram avaliadas utilizando o teste qui-quadrado ou o teste t de Student para amostras independentes. A associação entre IAI e DHGNA foi analisada utilizando um modelo de regressão logística multivariada ponderada. O Modelo 1 não foi ajustado; o Modelo 2 foi ajustado para fatores demográficos básicos, incluindo idade, sexo, raça, nível de escolaridade, estado civil e PIR; o Modelo 3 foi ajustado adicionalmente para IMC, tabagismo, hipertensão, diabetes, DCV, nível de atividade física, ALT, AST e SIRI. Para explorar a potencial relação não linear entre IAI e DHGNA, um modelo de spline cúbico restrito (RCS) foi construído. Uma regressão linear em dois estágios foi aplicada adicionalmente para identificar pontos de inflexão. Análises de subgrupos foram conduzidas de acordo com sexo, idade, IMC, tabagismo, hipertensão, diabetes e nível de atividade física para avaliar a consistência das associações e potenciais interações. O valor diagnóstico do IAI para DHGNA foi avaliado utilizando curvas ROC (Receiver Operating Characteristic), e a área sob a curva (AUC) foi calculada. As diferenças entre as AUCs foram comparadas estatisticamente utilizando o teste de DeLong para avaliar as diferenças de desempenho na distinção entre DHGNA e não-DHGNA. Todas as análises estatísticas foram realizadas utilizando o software R versão 4.5.1. Um valor de p bicaudal < 0,05 foi considerado estatisticamente significativo.

## Resultados

### Características básicas dos participantes

Um total de 5.309 adultos participaram deste estudo, dos quais 1.996 (aproximadamente 37,6%) foram diagnosticados com DHGNA (Tabela 1). Em relação à distribuição por idade, a proporção de participantes de meia-idade e idosos (≥ 60 anos) foi maior no grupo com DHGNA do que no grupo sem DHGNA. A distribuição por sexo indicou que a proporção de homens foi maior no grupo com DHGNA em comparação com o grupo sem DHGNA. Observou-se uma diferença significativa no IMC entre os grupos: a proporção de indivíduos obesos (IMC ≥ 30 kg/m^2^) foi maior no grupo com DHGNA, enquanto a proporção de participantes com peso normal (IMC < 25 kg/m^2^) foi menor em comparação com o grupo sem DHGNA. Quanto à distribuição racial, a proporção de mexicano-americanos foi maior, enquanto a de negros não hispânicos foi menor no grupo com DHGNA. A relação entre escolaridade e renda familiar abaixo da linha da pobreza (PIR) foi semelhante entre os dois grupos, enquanto a proporção de participantes casados foi ligeiramente maior no grupo com DHGNA. As características de estilo de vida indicaram que a proporção de fumantes foi maior no grupo com DHGNA, enquanto a proporção de participantes que praticavam atividade física foi menor em comparação com o grupo sem DHGNA. Comorbidades relacionadas ao metabolismo, incluindo diabetes, hipertensão e doenças cardiovasculares, foram mais prevalentes no grupo com DHGNA. Os indicadores laboratoriais revelaram que tanto o IAI quanto o SIRI foram maiores no grupo com DHGNA em comparação com o grupo sem DHGNA. Os testes de função hepática mostraram níveis elevados de AST e ALT no grupo com DHGNA, com diferenças significativas em comparação com o grupo sem DHGNA (p < 0,001).

**Tabela 1:** – Características dos participantes agrupados pela presença ou ausência de DHGNA

Características	Total (n=5.309)	DHGNA (n=1.996)	Não-DHGNA (n=3.313)	Valor de p
**Idade (anos)**				<0,001
<60	3508 (66,1%)	1254 (62,8%)	2254 (68,0%)	
≥60	1801 (33,9%)	742 (37,2%)	1059 (32,0%)	
**Sexo, n(%)**				<0,001
Macho	2347 (44,2%)	1008 (50,5%)	1339 (40,4%)	
Fêmea	2962 (55,8%)	988 (49,5%)	1974 (59,6%)	
**IMC, n(%)**				<0,001
<25 kg/m2	1334 (25,1%)	118 (5,9%)	1216 (36,7%)	
25-30 kg/m^2^	1699 (32,0%)	533 (26,7%)	1166 (35,2%)	
≥30 kg/m2	2276 (42,9%)	1345 (67,4%)	931 (28,1%)	
**PIR, n(%)**				0,240
<1,3	1380 (26,0%)	510 (25,6%)	870 (26,3%)	
1,3–3,49	2070 (39,0%)	807 (40,4%)	1263 (38,1%)	
≥3,5	1859 (35,0%)	679 (34,0%)	1180 (35,6%)	
**Raça, n(%)**				<0,001
Mexicano-americano	626 (11,8%)	332 (16,6%)	294 (8,9%)	
Negro não hispânico	1309 (24,7%)	411 (20,6%)	898 (27,1%)	
Branco não hispânico	1899 (35,8%)	749 (37,5%)	1150 (34,7%)	
Outros hispânicos	526 (9,9%)	190 (9,5%)	336 (10,1%)	
Outro	949 (17,9%)	314 (15,7%)	635 (19,2%)	
**Educação, n(%)**				0,085
Abaixo do ensino médio	862 (16,2%)	347 (17,4%)	515 (15,5%)	
Acima do ensino médio	4447 (83,8%)	1649 (82,6%)	2798 (84,5%)	
**Casados, n(%)**				<0,001
Sim	3160 (59,5%)	1291 (64,7%)	1869 (56,4%)	
Não	2149 (40,5%)	705 (35,3%)	1444 (43,6%)	
**Tabagismo, n(%)**				<0,001
Sim	1910 (36,0%)	800 (40,1%)	1110 (33,5%)	
Não	3399 (64,0%)	1196 (59,9%)	2203 (66,5%)	
**DCV, n(%)**				0,002
Sim	535 (10,1%)	234 (11,7%)	301 (9,1%)	
Não	4774 (89,9%)	1762 (88,3%)	3012 (90,9%)	
**Atividade física,** n (%)				<0,001
Sim	2236 (42,1%)	762 (38,2%)	1474 (44,5%)	
Não	3073 (57,9%)	1234 (61,8%)	1839 (55,5%)	
**Diabetes, n(%)**				<0,001
Sim	1037 (19,5%)	649 (32,5%)	388 (11,7%)	
Não	4272 (80,5%)	1347 (67,5%)	2925 (88,3%)	
**Hipertensão, n (%)**				<0,001
Sim	2584 (48,7%)	1156 (57,9%)	1428 (43,1%)	
Não	2725 (51,3%)	840 (42,1%)	1885 (56,9%)	
**IAI**	-0,02 ± 1,31	0,59 ± 1,26	-0,39 ± 1,20	<0,001
**SIRI**	1,21 ± 0,90	1,26 ± 0,89	1,17 ± 0,90	<0,001
**AST (U/L)**	21,59 ± 15,58	26,50 ± 19,27	18,63 ± 11,92	<0,001
**ALT (U/L)**	21,22 ± 11,81	22,74 ± 14,66	20,31 ± 9,59	<0,001

PIR: índice de pobreza/renda familiar; IMC: índice de massa corporal; DCV: doença cardiovascular; AST: aspartato aminotransferase; ALT: alanina aminotransferase. IAI: índice de aterosclerose inflamatória; DHGNA: doença hepática gordurosa não alcoólica .

### A associação entre IAI e doença hepática gordurosa não alcoólica

A análise de regressão logística ponderada multivariada mostrou que o IAI estava significativamente associado a um risco aumentado de DHGNA (Tabela 2). No modelo não ajustado (Modelo 1), a razão de chances (RC) por aumento de uma unidade no IAI foi de 2,081 (IC 95%: 1,964–2,205, p < 0,001). Após ajuste adicional para múltiplos fatores de confusão potenciais no Modelo 3, a associação permaneceu significativa (RC = 1,538, IC 95%: 1,421–1,665, p < 0,001). A análise de quartis do IAI indicou que um IAI mais elevado estava associado a um risco progressivamente crescente de DHGNA. No Modelo 3, com o quartil mais baixo (Q1) como referência, o risco de DHGNA foi significativamente maior no Q4 (RC = 3,954, IC 95%: 2,740–5,707, p = 0,018). Essa tendência foi consistente em todos os três modelos (todos os valores de p para tendência < 0,001).

**Tabela 2:** – Análise de regressão logística ponderada multivariada de IAI e DHGNA

	Modelo 1	Modelo 2	Modelo 3

	OR (IC 95%) *valor p*	OR (IC 95%) *valor p*	OR (IC 95%) *valor p*
IAI	2,081 (1,964, 2,205) <0,001	2,062 (1,954, 2,176) <0,001	1,538 (1,421, 1,665) <0,001
IAI quartil			
1º trimestre < -0,95	1(ref.)	1(ref.)	1(ref.)
2º trimestre -0,95 ~-0,12	2,449 (1,749, 3,428) <0,001	2,382 (1,704, 3,328) <0,001	1,432 (0,987, 2,078) 0,200
3º trimestre -0,12 ~ 0,80	6,282 (4,673, 8,446) <0,001	6,046 (4,540, 8,052) <0,001	2,771 (2,044, 3,756) 0,022
4º trimestre > 0,80	11.830 (9.312, 15.029) <0,001	11.062 (8.766, 13.960) <0,001	3,954 (2,740, 5,707) 0,018
p para tendência	<0,001	<0,001	<0,001

Modelo 1: Nenhum ajuste foi feito; Modelo 2: Ajustado por sexo, idade, raça, escolaridade, PIR e estado civil; Modelo 3: Com base no Modelo 2, incorporar ainda índice de massa corporal, tabagismo, hipertensão, diabetes, doença cardiovascular, nível de atividade física, alanina aminotransferase, aspartato aminotransferase e SIRI. IAI: índice de aterosclerose inflamatória.

A relação entre o IAI e o risco de DHGNA foi analisada utilizando curvas de spline cúbica restrita (RCS) (Figura 2). Os resultados indicaram não linearidade significativa (P para não linearidade = 0,0473). A análise de regressão linear em dois estágios identificou um ponto de inflexão em 0,145. Para IAI < 0,145, o risco de DHGNA aumentou significativamente (OR = 2,341, IC 95%: 2,037–2,690, p < 0,001). Para IAI ≥ 0,145, o risco continuou a aumentar, embora com menor magnitude (OR = 1,399, IC 95%: 1,263–1,548, p < 0,001), conforme mostrado na Tabela 3.

**Figura 2:**
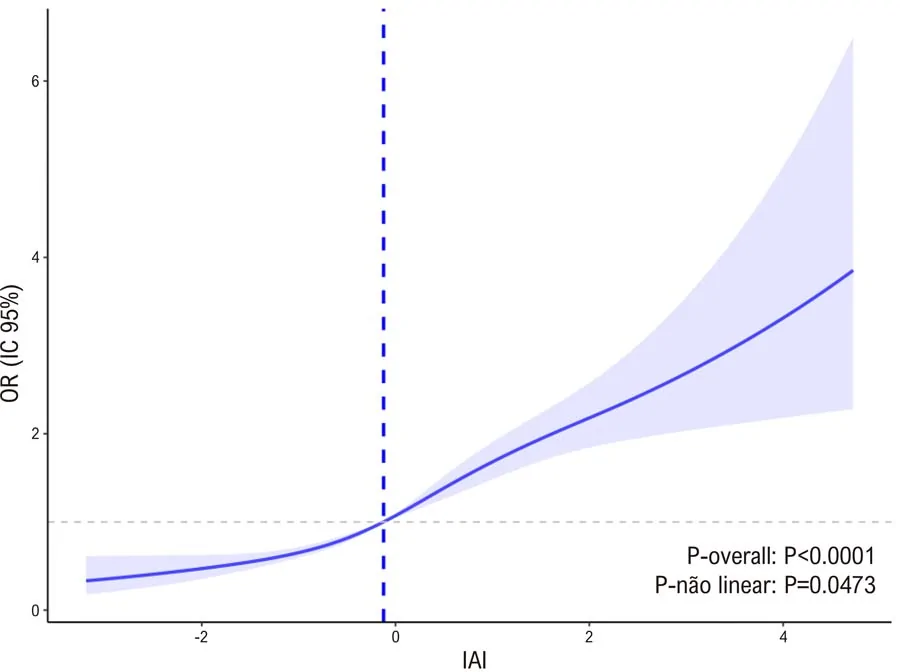
– Análise de spline cúbica restrita da associação entre IAI e DHGNA. A linha contínua indica a razão de chances ajustada e a área sombreada indica o intervalo de confiança de 95%. IAI: índice de aterosclerose inflamatória; DHGNA: doença hepática gordurosa não alcoólica.

OR (IC 95%)

**Tabela 3:** – Análise do efeito limiar da associação entre IAI e DHGNA

	OU (IC 95%)	Valor p
**Modelo de regressão linear segmentada de dois segmentos**		
O ponto de inflexão do IAI	0,145	
< 0.145	2,341 (2,037, 2,690)	<0,001
> 0,145	1,399 (1,263, 1,548)	<0,001
Razão de verossimilhança logarítmica		<0,001

IAI: índice de aterosclerose inflamatória.

### Análise de subgrupos

Para avaliar a consistência da associação entre IAI e DHGNA em diferentes subgrupos, realizamos uma análise de subgrupos (Figura 3). Os resultados mostraram que o IAI esteve significativamente associado a um risco aumentado de DHGNA em todas as categorias de idade, sexo, IMC, tabagismo e níveis de atividade física, bem como em participantes com ou sem diabetes, hipertensão ou doenças cardiovasculares (todos com p < 0,05). Não foram observadas interações estatisticamente significativas entre os subgrupos (p para interação > 0,05).

**Figura 3:**
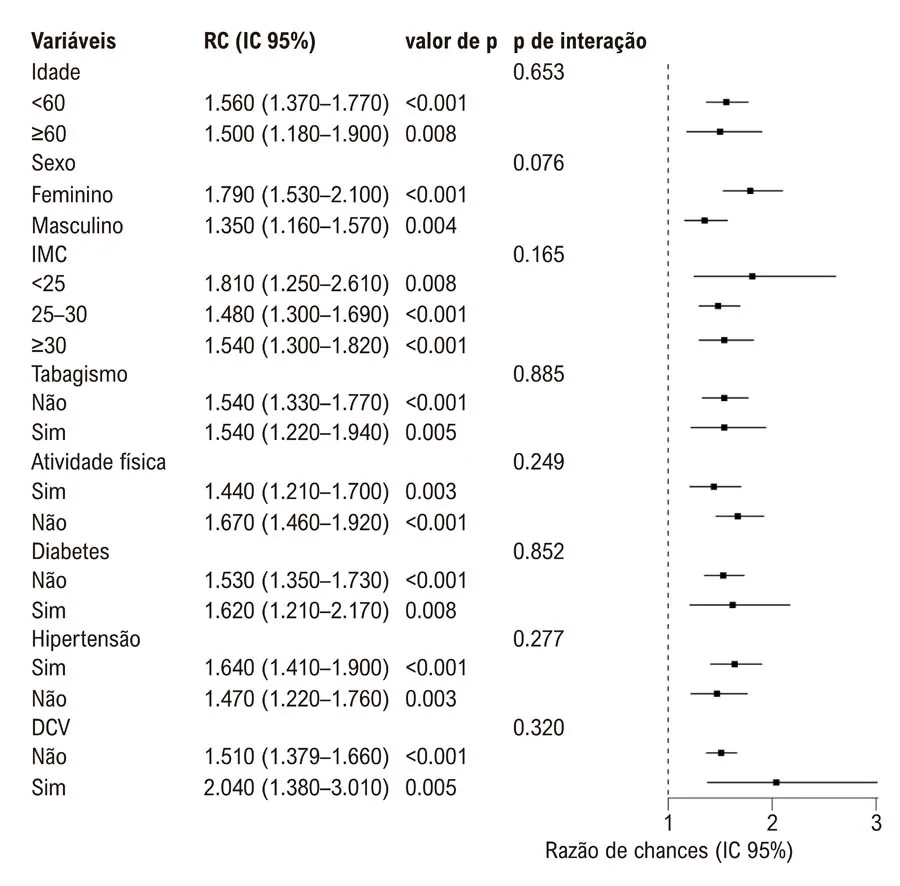
– Análises de subgrupos da associação entre IAI e DHGNA. Razões de chances (odds ratios) e intervalos de confiança de 95% são mostrados em todos os subgrupos. IAI: índice de aterosclerose inflamatória; DHGNA: doença hepática gordurosa não alcoólica.

### O valor diagnóstico do IAI

A análise da curva ROC (Figura 4) mostrou que o IAI apresentou a maior eficácia diagnóstica para DHGNA (AUC = 0,722), superando a dos triglicerídeos (TG, AUC = 0,701), do colesterol HDL (HDL-C, AUC = 0,688) e da proteína C-reativa de alta sensibilidade (PCR-as, AUC = 0,549). Todos os testes de DeLong foram estatisticamente significativos, sugerindo que, como um indicador que reflete de forma abrangente o metabolismo lipídico e o estado inflamatório, o IAI possui valor diagnóstico superior na distinção entre pacientes com DHGNA e participantes sem DHGNA.

**Figura 4:**
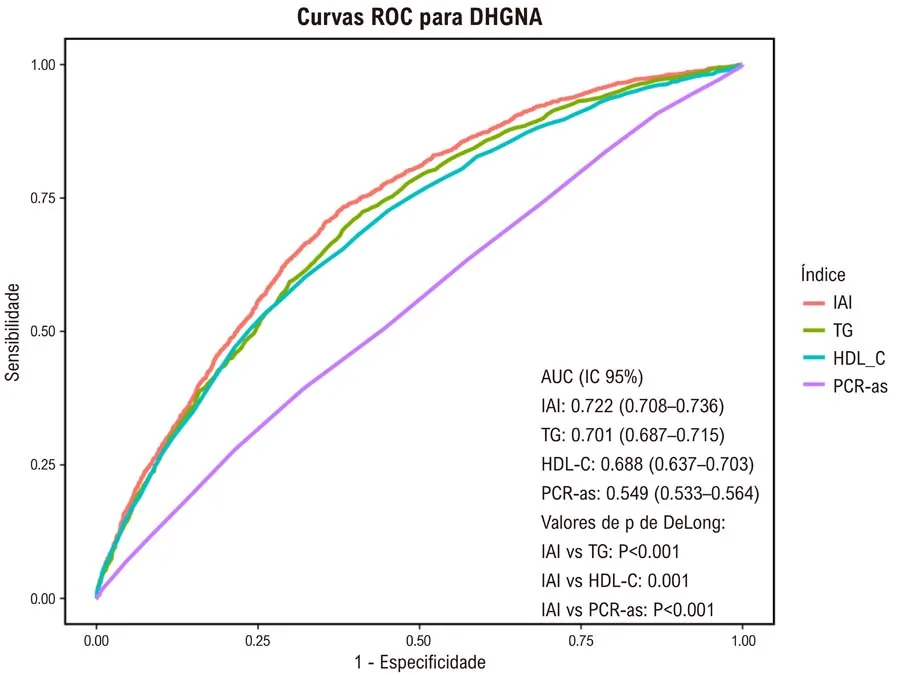
Curvas ROC (Receiver Operating Characteristic) do IAI e seus indicadores componentes para identificação de DHGNA. IAI: índice de aterosclerose inflamatória; TG: triglicerídeos; HDL-C: colesterol de lipoproteína de alta densidade; PCR-as: proteína C-reativa de alta sensibilidade; DHGNA: doença hepática gordurosa não alcoólica.

## Discussão

Este estudo utilizou dados representativos a nível nacional do NHANES nos Estados Unidos para avaliar sistematicamente a associação entre o IAI e o risco de DHGNA. Os resultados indicaram que o IAI estava significativamente elevado em pacientes com DHGNA e permaneceu consistentemente um fator de risco independente para DHGNA em modelos multivariados. O IAI exibiu um padrão de risco crescente estável em comparações interquartis. A análise por spline cúbico restrito (RCS) indicou ainda que a relação entre o IAI e o risco de DHGNA não era simplesmente linear, mas sim uma associação não linear significativa, com um ponto de inflexão de risco em IAI = 0,145. De acordo com a literatura anterioPara valores de IAI abaixo desse limiar, o risco aumentou acentuadamente com o aumento do IAI. Para valores acima do limiar, o aumento diminuiu, indicando maior sensibilidade para a identificação precoce do risco.

Do ponto de vista do mecanismo patológico, a ocorrência e a progressão da DHGNA envolvem um processo sequencial de acúmulo de lipídios,^19^ lipotoxicidade^20^ e ativação imunológica^21^ Pesquisas básicas indicaram que o acúmulo de ácidos graxos livres nos hepatócitos pode desencadear estresse no retículo endoplasmático, disfunção mitocondrial e produção de espécies reativas de oxigênio.^22^ Entretanto, o estresse dos hepatócitos pode ativar células imunes infiltrantes e induzir a liberação de fatores inflamatórios, estabelecendo assim um ciclo de retroalimentação positiva de dano metabólico e amplificação inflamatória.^23^ O IAI integra componentes de triglicerídeos que refletem a carga lipídica com indicadores relacionados à inflamação em um índice composto, capaz de capturar anormalidades sistêmicas concomitantes no metabolismo lipídico e na inflamação. Este estudo demonstrou que, em comparação com um único indicador metabólico ou inflamatório, a associação entre o IAI e o risco de DHGNA foi mais forte, o que está de acordo com a observação clínica de que pacientes com DHGNA frequentemente apresentam dislipidemia e estado inflamatório elevado.

Vale ressaltar que o IAI apresenta uma relação não linear com o risco de DHGNA, com um ponto de inflexão em aproximadamente 0,145. Para valores de IAI abaixo desse ponto de inflexão, cada aumento de uma unidade tem um efeito mais pronunciado no risco de DHGNA. No entanto, uma vez ultrapassado o ponto de inflexão, o risco continua a aumentar, embora a uma taxa mais lenta. Esse padrão é consistente com o estágio inicial de vulnerabilidade metabólica da DHGNA. Durante a ruptura inicial da homeostase metabólica, o fígado é altamente sensível à inflamação leve e às anormalidades lipídicas, e mesmo pequenas perturbações podem desencadear o acúmulo de gotículas lipídicas hepáticas e danos hepatocelulares precoces. Após entrar em um estágio mais estável de metabolismo inflamatório anormal, o aumento do risco se atenua.^24-26^ Efeitos de “limiar” semelhantes foram observados em estudos de doenças metabólicas, como a resistência à insulina.^27^e inflamação do tecido adiposo.^28^ Este estudo fornece evidências quantitativas que sugerem que a IAI pode ter maior valor para a triagem precoce de risco de DHGNA.

Este estudo apresenta vários pontos fortes. Primeiro, baseia-se em uma grande amostra nacionalmente representativa do NHANES, proporcionando um alto grau de generalização externa. Segundo, potenciais fatores de confusão — incluindo características demográficas, estilo de vida, comorbidades metabólicas e múltiplos indicadores relacionados à inflamação — foram sistematicamente integrados, aumentando a robustez das estimativas de associação. Terceiro, este estudo é o primeiro a avaliar sistematicamente a relação entre o IAI e a DHGNA em nível populacional. Diversas abordagens estatísticas, incluindo análise de quartis, splines cúbicos restritos e análises de subgrupos, foram empregadas para revelar as características não lineares e os padrões de ponto de inflexão, oferecendo novas ferramentas e perspectivas para a identificação de risco de DHGNA e pesquisas epidemiológicas relacionadas. A consistência da associação entre múltiplos subgrupos reforça ainda mais a confiabilidade dos achados.

Este estudo também apresenta algumas limitações. Primeiro, o NHANES é um estudo transversal, o que impossibilita determinar se o aumento da IAI precede o início da DHGNA; portanto, as relações causais precisam ser confirmadas por meio de estudos prospectivos. Segundo, o padrão ouro para o diagnóstico de DHGNA é a biópsia hepática histológica, enquanto este estudo utilizou um método diagnóstico não considerado padrão ouro, o que pode introduzir erros de classificação. Além disso, o conjunto de dados carece de informações metabólicas detalhadas, como composição da dieta, uso de medicamentos e microbiota intestinal, o que pode contribuir para fatores de confusão residuais. Estudos futuros que integrem delineamentos de coorte prospectivos com dados multiômicos — incluindo metabolômica, lipidômica e perfil inflamatório — ajudarão a elucidar ainda mais os mecanismos biológicos que ligam a IAI à DHGNA e a verificar sua potencial utilidade na predição de risco e intervenção.

## Conclusão

Este estudo, baseado em dados populacionais de grande escala, demonstra sistematicamente uma associação positiva independente entre o IAI e a DHGNA. Os resultados indicam uma relação não linear entre os níveis de IAI e o risco de DHGNA. Essa associação permanece consistente em diferentes faixas etárias, sexos, estilos de vida e subgrupos definidos por comorbidades metabólicas. Além disso, o IAI apresenta melhor desempenho do que indicadores metabólicos isolados no diagnóstico de DHGNA, sugerindo considerável utilidade clínica como um marcador abrangente de inflamação e metabolismo. No entanto, devido às limitações do delineamento transversal, esses achados requerem confirmação em estudos prospectivos.
