# Supplementary material for: Associação entre o Índice de Aterosclerose Inflamatória e a Doença Hepática Gordurosa Não Alcoólica: NHANES 2017–2020
Source: Arq Bras Cardiol. 2026 Oct 21;123(9):e20260068. [Article in Portuguese] doi: 10.36660/abc.20260068 (PMC13754397; doi:10.36660/abc.20260068)
Supplement: Translation

# Translation: Association between the Inflammatory Atherosclerosis Index and Non-Alcoholic Fatty Liver Disease: NHANES 2017–2020

**DOI:** 10.36660/abc.20260068I

**Authors:** Yun Jiang, Jingying Xie, Ying Dong

**Affiliations:** 1 China Department of Hepatobiliary and Pancreatic Surgery II, Ningbo Medical Centre Lihuili Hospital, Zhejiang Province, Ningbo – China

**Keywords:** Non-alcoholic Fatty Liver Disease, Nutrition Surveys, Cross-Sectional Studies

## Abstract

**Background:**

The Non-alcoholic fatty liver disease (NAFLD) prevalence is increasing globally and is closely associated with dysregulated lipid metabolism and inflammation. However, the relationship between NAFLD and the inflammatory atherosclerosis index (IAI), which reflects these processes, is unclear.

**Objectives:**

To investigate the association between IAI and NAFLD in a representative sample of U.S. adults.

**Methods:**

This cross-sectional study included 5,309 adults from the U.S. NHANES database. Multivariate weighted logistic regression was used to assess the association between IAI and NAFLD. Restricted cubic splines explored nonlinear relationships, and ROC analysis evaluated diagnostic performance.

**Results:**

A total of 5,309 participants were included, of whom 37.6% had NAFLD. Obesity and metabolic abnormalities were more pronounced in the NAFLD group, and IAI levels were significantly higher (P < 0.001). Multivariate analysis demonstrated that IAI was independently and positively associated with NAFLD (OR = 1.538, 95% CI: 1.421–1.665). RCS analysis indicated a nonlinear relationship (P for nonlinearity = 0.0473), with an inflection point at IAI = 0.145. When IAI < 0.145, the risk increased steeply (OR = 2.341, 95% CI: 2.037–2.690), whereas when IAI ≥ 0.145, the increase was attenuated (OR = 1.399, 95% CI: 1.263–1.548). Subgroup analyses showed consistent results, with no significant interactions across subgroups. ROC analysis showed that IAI exhibited the best diagnostic performance (AUC = 0.722), significantly outperforming other indicators (all P < 0.001).

**Conclusions:**

Among U.S. adults, IAI demonstrates a strong, positive, and nonlinear association with NAFLD. These findings suggest IAI is a promising population-level marker for NAFLD, though prospective studies are required for validation.

## Introduction

Non-alcoholic fatty liver disease (NAFLD) is one of the most common chronic liver diseases globally. The prevalence of NAFLD has risen in parallel with increasing rates of obesity, type 2 diabetes, and metabolic syndrome, posing a major global public health challenge.^1-3^ The pathophysiology of NAFLD is complex and involves multiple factors, including dysregulated lipid metabolism, insulin resistance, chronic low-grade inflammation, and oxidative stress.^4,5^ As NAFLD advances, a subset of patients develop non-alcoholic steatohepatitis (NASH), liver fibrosis, cirrhosis, or hepatocellular carcinoma. Early identification of high-risk individuals and associated risk factors is therefore critical for disease management, risk stratification, and the design of intervention strategies.

Inflammation and dysregulated lipid metabolism are regarded as central mechanisms driving the onset and progression of NAFLD.^6,7^ Chronic low-grade inflammation promotes hepatic lipid accumulation and hepatocyte injury via pro-inflammatory cytokines and oxidative-stress-mediated pathways. Meanwhile, dyslipidemia can exacerbate hepatic fat accumulation and metabolic stress. This crosstalk between inflammation and lipid metabolism not only influences hepatic pathology but may also increase cardiovascular and systemic metabolic risk.^8,9^ Therefore, indicators that integrate inflammatory and lipid information are valuable for epidemiological studies of NAFLD.

The Inflammatory Atherosclerosis Index (IAI) is a composite measure that integrates lipid parameters and systemic inflammatory burden.^10^ The IAI combines lipid measurements with high-sensitivity C-reactive protein (hs-CRP) to quantify dyslipidemia and systemic inflammatory burden, thereby reflecting overall metabolic stress. Higher IAI values indicate greater lipid abnormalities and inflammatory burden, reflecting heightened inflammation-related metabolic stress and serving as a potential biomarker for metabolism-related diseases.

Although previous studies have linked dyslipidemia and inflammatory status to NAFLD,^11,12^ few systematic analyses have evaluated composite indices such as the IAI. Therefore, we systematically evaluated the association between the IAI and NAFLD using the nationally representative NHANES 2017–2020 sample. We aimed to provide empirical evidence for early risk identification and characterization of metabolic features of NAFLD, and to inform the potential use of liver-function and inflammation-related markers for disease prediction in non-tumor populations.

## Materials and methods

### Data collection

This study performed a cross-sectional analysis using data from the NHANES 2017–2020 cycles. NHANES is a nationally representative health and nutrition survey in the United States that is conducted biennially. It collects information on participants’ health and nutritional status through questionnaires, physical examinations, and laboratory tests. The initial sample included 15,560 participants. To ensure an appropriate study population and complete data, we excluded participants younger than 20 years, those who tested positive for hepatitis B surface antigen or hepatitis C antibody/RNA, individuals who self-reported excessive alcohol consumption (≥ 4–5 drinks per day), and those with missing covariate data. A total of 5,309 eligible adults were ultimately included in the final analytic sample. The data cleaning and screening process is presented (Figure 1 and Central Illustration).

**Figure 1:** – Flowchart of participant selection from NHANES 2017–2020. NHANES: National Health and Nutrition Examination Survey; PIR: poverty income ratio; HBV: hepatitis B virus; HCV: hepatitis C virus; CVD: cardiovascular diseases; ALT: alanine aminotransferase; AST: aspartate aminotransferase; BMI: body mass index.

### The definition of non-alcoholic fatty liver disease

The determination of NAFLD is based on the controlled attenuation parameter (CAP) obtained from liver transient elastography. According to previous literature,^13^ individuals with a CAP value greater than 285 dB/m were classified as having NAFLD, while all others were assigned to the non-NAFLD group. This criterion provides a reliable and non-invasive method for assessing hepatic fat accumulation.

### Inflammatory atherosclerosis index

The IAI is a composite indicator that comprehensively reflects lipid metabolism abnormalities and systemic inflammatory burden and is used to capture the interplay between dyslipidemia and systemic inflammation.^14^ The IAI is calculated as: IAI = log_10_ (triglycerides / high-density lipoprotein cholesterol) × hs-CRP / 10. Higher IAI values indicate greater lipid metabolism abnormalities and a higher systemic inflammatory burden.

### Covariate

This study included gender, age, race/ethnicity, educational level, marital status, household poverty-income ratio (PIR), body mass index (BMI), smoking, hypertension, diabetes, cardiovascular disease (CVD), physical activity level, alanine aminotransferase (ALT), aspartate aminotransferase (AST), and the systemic inflammatory response index (SIRI) as covariates. The determination of diabetes was based on self-reported diagnosis, the use of insulin or oral hypoglycemic agents, and laboratory indicators (fasting blood glucose ≥ 7.0 mmol/L or HbA1c ≥ 6.5%).^15^ CVD includes coronary heart disease, angina pectoris, myocardial infarction, heart failure, and stroke. The information was obtained from the NHANES Medical History Questionnaire.^16^ Hypertension was defined as an average systolic blood pressure ≥ 140 mmHg, an average diastolic blood pressure ≥ 90 mmHg, or self-reported diagnosis or use of antihypertensive drugs.^17^ ALT, AST, and SIRI were obtained from the NHANES laboratory measurements. SIRI was calculated as monocytes × neutrophils ÷ lymphocytes.^18^

### Statistical analysis

This study fully accounted for the complex NHANES sampling design, including stratification, clustering, and multi-stage sampling. Survey weighting was applied to ensure the national representativeness and generalizability of the results. The baseline characteristics of the participants were described according to NAFLD status. Continuous variables were presented as mean ± standard deviation (SD), and categorical variables as frequency and percent. The differences between groups were evaluated using the chi-square test or the independent-samples t-test. The association between IAI and NAFLD was analyzed using a weighted multivariate logistic regression model. Model 1 was unadjusted; Model 2 adjusted for basic demographic factors, including age, gender, race, education level, marital status, and PIR; Model 3 further adjusted for BMI, smoking, hypertension, diabetes, CVD, physical activity level, ALT, AST, and SIRI. To explore the potential nonlinear relationship between IAI and NAFLD, a restricted cubic spline (RCS) model was constructed. A two-stage linear regression was additionally applied to identify inflection points. Subgroup analyses were conducted according to gender, age, BMI, smoking, hypertension, diabetes, and physical activity level to assess the consistency of associations and potential interactions. The diagnostic value of IAI for NAFLD was evaluated using receiver operating characteristic (ROC) curves, and the area under the curve (AUC) was calculated. Differences among AUCs were statistically compared using the DeLong test to assess performance differences in distinguishing NAFLD from non-NAFLD. All statistical analyses were performed using R version 4.5.1. A two-sided p-value < 0.05 was considered statistically significant.

## Results

### Basic characteristics of participants

A total of 5,309 adults were included in this study, of whom 1,996 (approximately 37.6%) were diagnosed with NAFLD (Table 1). Regarding age distribution, the proportion of middle-aged and elderly participants (≥ 60 years) was higher in the NAFLD group than in the non-NAFLD group. The gender distribution indicated that the proportion of males was higher in the NAFLD group compared with the non-NAFLD group. A significant difference in BMI was observed between the groups: the proportion of obese individuals (BMI ≥ 30 kg/m^2)^ was higher in the NAFLD group, whereas the proportion of participants with normal weight (BMI < 25 kg/m^2^) was lower compared with the non-NAFLD group. Regarding racial distribution, the proportion of Mexican Americans was higher, while that of non-Hispanic Blacks was lower in the NAFLD group. The ratio of educational attainment to household poverty income (PIR) was similar between the two groups, whereas the proportion of married participants was slightly higher in the NAFLD group. Lifestyle characteristics indicated that the proportion of smokers was higher in the NAFLD group, whereas the proportion of participants engaging in physical activity was lower compared with the non-NAFLD group. Metabolism-related comorbidities, including diabetes, hypertension, and cardiovascular diseases, were more prevalent in the NAFLD group. Laboratory indicators revealed that both IAI and SIRI were higher in the NAFLD group compared with the non-NAFLD group. Liver function tests showed elevated AST and ALT levels in the NAFLD group, with significant differences compared to the non-NAFLD group (p < 0.001).

**Table 1:** – Characteristics of participants grouped by the presence or absence of NAFLD

Characteristics	Total (n=5,309)	NAFLD (n=1,996)	No-NAFLD (n=3,313)	p value
**Age (years)**				<0.001
<60	3508 (66.1%)	1254 (62.8%)	2254 (68.0%)	
≥60	1801 (33.9%)	742 (37.2%)	1059 (32.0%)	
**Gender, n(%)**				<0.001
Male	2347 (44.2%)	1008 (50.5%)	1339 (40.4%)	
Female	2962 (55.8%)	988 (49.5%)	1974 (59.6%)	
**BMI, n(%)**				<0.001
<25 kg/m^2^	1334 (25.1%)	118 (5.9%)	1216 (36.7%)	
25-30 kg/m^2^	1699 (32.0%)	533 (26.7%)	1166 (35.2%)	
≥30 kg/m^2^	2276 (42.9%)	1345 (67.4%)	931 (28.1%)	
**PIR, n(%)**				0.240
<1.3	1380 (26.0%)	510 (25.6%)	870 (26.3%)	
1.3–3.49	2070 (39.0%)	807 (40.4%)	1263 (38.1%)	
≥3.5	1859 (35.0%)	679 (34.0%)	1180 (35.6%)	
**Race, n(%)**				<0.001
Mexican American	626 (11.8%)	332 (16.6%)	294 (8.9%)	
Non-Hispanic Black	1309 (24.7%)	411 (20.6%)	898 (27.1%)	
Non-Hispanic White	1899 (35.8%)	749 (37.5%)	1150 (34.7%)	
Other Hispanic	526 (9.9%)	190 (9.5%)	336 (10.1%)	
Other	949 (17.9%)	314 (15.7%)	635 (19.2%)	
**Education, n(%)**				0.085
Below high school	862 (16.2%)	347 (17.4%)	515 (15.5%)	
Above high school	4447 (83.8%)	1649 (82.6%)	2798 (84.5%)	
**Married, n(%)**				<0.001
Yes	3160 (59.5%)	1291 (64.7%)	1869 (56.4%)	
No	2149 (40.5%)	705 (35.3%)	1444 (43.6%)	
**Smoking, n(%)**				<0.001
Yes	1910 (36.0%)	800 (40.1%)	1110 (33.5%)	
No	3399 (64.0%)	1196 (59.9%)	2203 (66.5%)	
**CVD, n(%)**				
Yes	535 (10.1%)	234 (11.7%)	301 (9.1%)	0.002
No	4774 (89.9%)	1762 (88.3%)	3012 (90.9%)	
**Physical activity,** n (%)				<0.001
Yes	2236 (42.1%)	762 (38.2%)	1474 (44.5%)	
No	3073 (57.9%)	1234 (61.8%)	1839 (55.5%)	
**Diabetes, n(%)**				<0.001
Yes	1037 (19.5%)	649 (32.5%)	388 (11.7%)	
No	4272 (80.5%)	1347 (67.5%)	2925 (88.3%)	
**Hypertension, n (%)**				<0.001
Yes	2584 (48.7%)	1156 (57.9%)	1428 (43.1%)	
No	2725 (51.3%)	840 (42.1%)	1885 (56.9%)	
**IAI**	-0.02 ± 1.31	0.59 ± 1.26	-0.39 ± 1.20	<0.001
**SIRI**	1.21 ± 0.90	1.26 ± 0.89	1.17 ± 0.90	<0.001
**AST (U/L)**	21.59 ± 15.58	26.50 ± 19.27	18.63 ± 11.92	<0.001
**ALT (U/L)**	21.22 ± 11.81	22.74 ± 14.66	20.31 ± 9.59	<0.001

NAFLD: non-alcoholic fatty liver disease; BMI: body mass index; PIR: poverty-income ratio; CVD: cardiovascular disease; IAI: inflammatory atherosclerosis index; SIRI: systemic inflammatory response index; AST: aspartate aminotransferase; ALT: alanine aminotransferase.

### The association between IAI and non-alcoholic fatty liver disease

Multivariate weighted logistic regression analysis showed that IAI was significantly associated with an increased risk of NAFLD (Table 2). In the unadjusted model (Model 1), the odds ratio (OR) per unit increase in IAI was 2.081 (95% CI: 1.964–2.205, p < 0.001). After further adjustment for multiple potential confounding factors in Model 3, the association remained significant (OR = 1.538, 95% CI: 1.421–1.665, p < 0.001). Quartile analysis of IAI indicated that higher IAI was associated with a gradually increasing risk of NAFLD. In Model 3, with the lowest quartile (Q1) as the reference, the risk of NAFLD was significantly higher in Q4 (OR = 3.954, 95% CI: 2.740–5.707, p = 0.018). This trend was consistent across all three models (all p for trend < 0.001).

**Table 2:** – Multivariate weighted Logistic regression analysis of IAI and NAFLD

	Model 1	Model 2	Model 3

	OR (95%CI) p-value	OR (95%CI) p-value	OR (95%CI) p-value
IAI	2.081 (1.964, 2.205) <0.001	2.062 (1.954, 2.176) <0.001	1.538 (1.421, 1.665) <0.001
IAI quartile			
Q1 < -0.95	1(ref.)	1(ref.)	1(ref.)
Q2 -0.95 ~ -0.12	2.449 (1.749, 3.428) <0.001	2.382 (1.704, 3.328) <0.001	1.432 (0.987, 2.078) 0.200
Q3 -0.12 ~ 0.80	6.282 (4.673, 8.446) <0.001	6.046 (4.540, 8.052) <0.001	2.771 (2.044, 3.756) 0.022
Q4 > 0.80	11.830 (9.312, 15.029) <0.001	11.062 (8.766, 13.960) <0.001	3.954 (2.740, 5.707) 0.018
p for trend	<0.001	<0.001	<0.001

Model 1: No adjustments have been made; Model 2: Adjust for gender, age, race, education, PIR, and marital status; Model 3: Based on Model 2, further incorporate body mass index, smoking, hypertension, diabetes, cardiovascular disease, physical activity level, alanine aminotransferase, aspartate aminotransferase, and SIRI. IAI: inflammatory atherosclerosis index.

The relationship between IAI and NAFLD risk was analyzed using restricted cubic spline (RCS) curves (Figure 2). The results indicated significant nonlinearity (P for nonlinearity = 0.0473). Two-stage linear regression analysis identified an inflection point at 0.145. For IAI < 0.145, the risk of NAFLD significantly increased (OR = 2.341, 95% CI: 2.037–2.690, p < 0.001). For IAI ≥ 0.145, the risk continued to increase, albeit with a smaller magnitude (OR = 1.399, 95% CI: 1.263–1.548, p < 0.001), as shown in Table 3.

**Figure 2:** – Restricted cubic spline analysis of the association between IAI and NAFLD. The solid line indicates the adjusted odds ratio, and the shaded area indicates the 95% confidence interval. IAI: inflammatory atherosclerosis index; NAFLD: non-alcoholic fatty liver disease.

OR (95% CI)

**Table 3:** – Threshold effect analysis of the association between IAI and NAFLD

	OR (95%CI)	p-value
**Two—piecewise linear regression model**		
The inflection point of IAI	0.145	
< 0.145	2.341 (2.037, 2.690)	<0.001
> 0.145	1.399 (1.263, 1.548)	<0.001
Log-likelihood ratio		<0.001

IAI: inflammatory atherosclerosis index.

### Subgroup analysis

To evaluate the consistency of the association between IAI and NAFLD across different subgroups, we conducted a subgroup analysis (Figure 3). The results showed that IAI was significantly associated with an increased risk of NAFLD across all categories of age, gender, BMI, smoking status, and physical activity levels, as well as in participants with or without diabetes, hypertension, or cardiovascular diseases (all p < 0.05). No statistically significant interactions were observed among the subgroups (p for interaction > 0.05).

**Figure 3:** – Subgroup analyses of the association between IAI and NAFLD. Odds ratios and 95% confidence intervals are shown across subgroups. IAI: inflammatory atherosclerosis index; NAFLD: non-alcoholic fatty liver disease.

### The diagnostic value of IAI

ROC curve analysis (Figure 4) showed that IAI had the highest diagnostic efficacy for NAFLD (AUC = 0.722), exceeding that of triglycerides (TG, AUC = 0.701), high-density lipoprotein cholesterol (HDL-C, AUC = 0.688), and high-sensitivity C-reactive protein (HsCRP, AUC = 0.549). All DeLong tests were statistically significant, suggesting that, as an indicator comprehensively reflecting lipid metabolism and inflammatory status, IAI has superior diagnostic value in distinguishing NAFLD patients from non-NAFLD participants.

**Figure 4:** – Receiver operating characteristic curves of IAI and its component indicators for identifying NAFLD. IAI: inflammatory atherosclerosis index; TG: triglycerides; HDL-C: high-density lipoprotein cholesterol; hs-CRP: high-sensitivity C-reactive protein; NAFLD: non-alcoholic fatty liver disease.

## Discussion

This study utilized nationally representative data from NHANES in the United States to systematically assess the association between IAI and the risk of NAFLD. The results indicated that IAI was significantly elevated in patients with NAFLD and remained consistently an independent risk factor for NAFLD in multivariate models. IAI exhibited a stable increasing risk pattern in interquartile comparisons. RCS analysis further indicated that the relationship between IAI and NAFLD risk was not simply linear, but a significant nonlinear association was observed, with a risk inflection point at IAI = 0.145. For IAI values below this threshold, the risk increased sharply with rising IAI. For values above the threshold, the increase slowed, indicating greater sensitivity for early risk identification.

From a pathological mechanism perspective, the occurrence and progression of NAFLD involves a sequential process of lipid accumulation,^19^ lipotoxicity,^20^ and immune activation.^21^ Basic research has indicated that the accumulation of free fatty acids within hepatocytes can trigger endoplasmic reticulum stress, mitochondrial dysfunction, and reactive oxygen species production.^22^ Meanwhile, hepatocyte stress may activate infiltrating immune cells and induce the release of inflammatory factors, thereby establishing a positive feedback loop of metabolic damage and inflammatory amplification.^23^ IAI integrates triglyceride components reflecting lipid load with inflammation-related indicators into a composite index, which can capture concurrent systemic abnormalities in lipid metabolism and inflammation. This study demonstrated that, compared with a single metabolic or inflammatory indicator, the association between IAI and NAFLD risk was stronger, consistent with the clinical observation that NAFLD patients commonly present with dyslipidemia and elevated inflammatory status.

It is worth noting that IAI exhibits a nonlinear relationship with the risk of NAFLD, with an inflection point occurring at approximately 0.145. For IAI values below this inflection point, each unit increase has a more pronounced effect on the risk of NAFLD. However, once the inflection point is exceeded, the risk continues to increase, albeit at a slower rate. This pattern is consistent with the early metabolic vulnerable stage of NAFLD. During the initial disruption of metabolic homeostasis, the liver is highly sensitive to mild inflammation and lipid abnormalities, and even minor disturbances can trigger hepatic lipid droplet accumulation and early hepatocellular damage. After entering a more stable stage of abnormal inflammatory metabolism, the increase in risk attenuates.^24-26^ Similar “threshold effects” have been observed in studies of metabolic diseases, such as insulin resistance^27^ and adipose tissue inflammation.^28^ This study provides quantitative evidence suggesting that IAI may have greater value for early risk screening of NAFLD.

This study has several strengths. First, it is based on a nationally representative large sample from NHANES, providing a high degree of external generalizability. Second, potential confounding factors—including demographic characteristics, lifestyle, metabolic comorbidities, and multiple inflammation-related indicators—were systematically integrated, enhancing the robustness of the association estimates. Third, this study is the first to systematically evaluate the relationship between IAI and NAFLD at the population level. Multiple statistical approaches, including quartile analysis, restricted cubic splines, and subgroup analyses, were employed to reveal the nonlinear characteristics and inflection point patterns, offering novel tools and perspectives for risk identification of NAFLD and related epidemiological research. The consistency of the association across multiple subgroups further supports the reliability of the findings.

This study also has certain limitations. First, NHANES is a cross-sectional survey, making it impossible to determine whether increases in IAI precede the onset of NAFLD; thus, causal relationships require confirmation through prospective studies. Second, the gold standard for diagnosing NAFLD is liver histological biopsy, whereas this study relied on a non-gold standard diagnostic method, introducing potential misclassification. Furthermore, the dataset lacks detailed metabolic information, such as dietary composition, medication use, and gut microbiota, which may contribute to residual confounding. Future studies integrating prospective cohort designs with multi-omics data—including metabolomics, lipidomics, and inflammatory profiling—will help further elucidate the biological mechanisms linking IAI and NAFLD and verify its potential utility in risk prediction and intervention.

## Conclusion

This study, based on large-scale population-based data, systematically demonstrates an independent positive association between IAI and NAFLD. The results indicate a nonlinear relationship between IAI levels and the risk of NAFLD. This association remains consistent across different age groups, sexes, lifestyles, and subgroups defined by metabolic comorbidities. Moreover, IAI outperforms single metabolic indicators in diagnosing NAFLD, suggesting considerable clinical utility as a comprehensive inflammation-metabolic marker. However, due to the limitations of the cross-sectional design, these findings require confirmation in prospective studies.
